# Dynamic Motile T Cells Highly Respond to the T Cell Stimulation via PI3K-Akt and NF-κB Pathways

**DOI:** 10.1371/journal.pone.0059793

**Published:** 2013-03-26

**Authors:** Hye-Ran Kim, Bo-Ra Na, Min-Sung Kwon, Yoo-Seung Ko, Weon-Cheol Han, Chang-Duk Jun

**Affiliations:** 1 School of Life Sciences, Immune Synapse Research Center and Cell Dynamics Research Center, Gwangju Institute of Science and Technology, Gwangju, Korea; 2 Department of Pathology, Wonkwang University School of Medicine, Iksan, Chonbuk, Korea; University of Maryland School of Medicine, United States of America

## Abstract

T lymphocytes (T cells) circulate from the blood into secondary lymphoid organs for immune surveillance. In this study, we hypothesized that circulating T cells are heterogeneous and can be grouped according to their differential migratory capacity in response to chemoattractants, rather than expressions of certain receptors or cytokines. We further hypothesized that, at least in part, this intrinsic difference in motility may be related to the T cell function. We established motile (m-T) and non-motile T (nm-T) cell lines based on their response to the chemokine SDF-1α. m-T cells showed more irregular and polarized morphologies than nm-T cells did. Interestingly, m-T cells produced higher levels of IL-2, a marker for T cell activation, than nm-T cells did after stimulation; however, no differences were observed in terms of surface expression of T cell receptors (TCR), adhesion molecules LFA-1 and ICAM-1, and chemokine receptor CXCR4. Both cell lines also showed similar membrane events (i.e., T cell-APC conjugation, LFA-1 accumulation at the immunological synapse, and TCR internalization). In contrast, PKC-θ, a downstream of PI3K-Akt pathway was constitutively activated in m-T cells and the activation was more prominent during T cell stimulation. Consequently, NF-κB activity was selectively upregulated in m-T cells. This study is the first, to our knowledge, to demonstrate that T cells can be subcategorized on the basis of their intrinsic migratory capacity in relation to T cell activation.

## Introduction

Lymphocytes are specialized migratory cells, continuously recirculating from the bloodstream into the secondary lymphoid organs (SLOs) and extravascular tissues for immune surveillance [1], [2], [3]. During infection with a pathogen, a series of events occur for the initiation of an immune response and elimination of the pathogen. The initial phase of the response is mediated by the recruitment of antigen-presenting cells (APCs), such as macrophages and dendritic cells. Activated APCs then migrate to lymphoid organs, and as a result, circulating naïve T cells first encounter the antigens on APCs in SLOs. This event stimulates naïve T cells to produce cytokines, which are required for clonal expansion and differentiation of naïve T cells into effector T cells. The migratory event of T lymphocytes is therefore a prerequisite and an indispensable process in triggering immune responses.

Trafficking of naïve T cells is controlled by a sequence of at least three molecularly distinct adhesion and signaling events [4], [5]. These adhesion cascades are initiated by a tethering step that allows leukocytes to bind loosely to endothelial cells. The marginated cells are then pushed forward in the blood stream, resulting in their slow rolling along the vessels (step 1). Subsequently, rolling cells encounter chemotactic stimuli on the endothelium that engage specific leukocyte receptors (step 2). Chemoattractant binding, in turn, induces intracellular signals, triggering activation-dependent adhesion steps that allow leukocytes to stick firmly together (step 3) and emigrate through the vessel wall.

During cell migration, lymphocytes obtain highly specialized motility and undergo morphological changes from round and symmetrical to a polarized and asymmetrical shape, because of chemokine-induced rapid actin polymerization and filament turnover [6]. The polarity of the T cells plays an important role in T cell sensitivity to antigens on APCs [7]. Thus, we hypothesized that circulating T cells are heterogeneous in terms of motility or polarity; therefore, they can be subcategorized according to their differential migratory capacities and different levels of sensitivities to chemoattractants. In addition, this intrinsic difference may be related to T cell functions. To this end, we established motile (m) and non-motile (nm) T cell lines, which show differential responses to chemokine stromal cell-derived factor-1α (SDF-1α).

The human chemokine system currently includes more than 50 chemokines, which can be classified by their cellular distribution and specific roles, e.g., “inflammatory chemokines for effector T cell function” and “homeostatic chemokines for naïve or memory T cells” [8]. Homeostatic chemokines are constitutively expressed, and they regulate the migration of lymphocytes and their precursors. Inflammatory chemokines are inducible, and they regulate the lymphocyte migration into tissues in response to an inflammatory stimulus, e.g., tissue damage, inflammation, or infection. In this study, because we aimed to determine whether there is any relationship between T cell activation and T cell migratory capacity in the condition that mimics the SLO-like environment, SDF-1α was chosen. This chemokine was chosen because it is a general homeostatic chemokine for naïve T cells [8], and most lymphocytes express CXCR4 (C-X-C chemokine receptor type 4), a SDF-1α receptor. In addition, SDF-1α induces by far the greatest lymphocyte transendothelial migration of the chemokines tested [9]. Therefore, we could establish cell lines on the basis of only a single parameter, i.e., cellular migratory capacity.

In this study, we utilized T cells that originated from 3 different sources, i.e., Jurkat T cells, human peripheral T cells, and mouse T cells. We characterized the features of m-T and nm-T cells and further determined that m-T cells exhibited elevated NF-κB activity through the PI3K-Akt signaling pathway and showed increased secretion of the cytokine IL-2 in response to the T-cell activation signals.

## Materials and Methods

### Cells and Mice

Jurkat T cells (ATCC TIB-152, Manassas, VA) and Raji B cells (ATCC number CCL-86, from Dr. Sánchez-Madrid F, Universidad Autonoma de Madrid, Madrid, Spain) were maintained in RPMI 1640 medium (GIBCO, Gaitherburg, MD) supplemented with 10% (v/v) FBS (GIBCO, Invitrogen). After written informed consent, human primary PBLs were isolated from healthy donors by dextran sedimentation and centrifugation through a discontinuous Ficoll gradient (Amersham Biosciences, Piscataway, NJ). The cell lines and human PBLs mentioned above were cultured at 37°C in a humidified incubator containing 5% CO_2_ and 95% air. All experiments using human PBLs were approved by Ethics Committee of the School of Life Sciences, GIST. C57BL/6 mice were housed under specific pathogen-free conditions. Mouse splenocytes were isolated from C57BL/6 and T lymphocytes, were enriched by using a CD3^+^ T cell enrichment column, and were cultured at 37°C in a humidified incubator containing 5% CO_2_ and 95% air. All the experiments were approved by the Animal Care and Use Committee of the School of Life Sciences, GIST.

### Reagent and Antibodies

PMA (phorbol 12-myristate 13-acetate), A23187, and PLL (poly-l-lysine) were from Sigma (St. Louis, MO). Staphylococcus Enterotoxin E (SEE) was obtained from Toxin Technology (Sarasota, FL) and hSDF-1α was purchased from Peprotech Inc. (Rocky Hill, NJ). PHA (phytohemagglutinin) and Con A (concanavalin A) were obtained from GIBCO (Gaitherburg, MD). Fluorescent dyes for cell labeling, green CMFDA (5-chloromethylfluorescein diacetate) and orange CMRA were from Molecular Probes (Invitrogen, Carlsbad, CA). Welprep Total RNA isolation reagent was purchased from JBI (Join Bio Innovation, Korea). Reverse transcript PCR premix, conventional PCR premix, and ECL western blotting detection reagents were from iNtRON Biotechnology (Seongnam-si, Korea). ImmunoPure Fab preparation kit was from Pierce and Cy-3 bisfunctional dye kit was from Amersham Biosciences (Piscataway, NJ). Mouse T cell enrichment column and DuoSet human IL-2 ELISA kit were purchased from R&D Systems (Minneapolis, MN). Anti-human CD28 was purchased from R&D Systems and FITC-conjugated CXCR4, and anti-mouse CD28 were purchased from BD Biosciences (San Jose, CA). OKT3 (human anti-CD3; CRL-8001) hybridoma cell lines were purchased from ATCC. TS1/18 (anti-human LFA-1; HB-203) and R6.5 (anti-human ICAM-1; ATCC HB-9580) hybridoma cell lines were gifted by Dr. T. A. Springer (Harvard Medical School). Rabbit polyclonal anti-phospho-ZAP70, rabbit anti- ZAP70 (99F2), rabbit polyclonal anti-SLP76, rabbit polyclonal anti-phospho-SLP76, rabbit anti-p44/42 MAPK (137F5), rabbit anti-phospho-p44/42 (197G2), HRP-conjugated anti-mouse IgG, and anti-rabbit IgG were purchased from Cell Signaling Technology (Beverly, MA). Goat polyclonal anti-β-actin, rabbit polyclonal anti-I-κB, rabbit polyclonal anti-phospho PI3K, mouse polyclonal anti-phospho Akt, mouse polyclonal anti-phospho PKC-θ, and rabbit polyclonal anti- PKC-θ were purchased from Santa Cruz Biotechnology (Santa Cruz, CA). Phalloidin-TRITC, secondary antibodies including FITC-conjugated anti-rabbit IgG, anti-goat IgG, and anti-hamster IgG were purchased from Sigma. PE-conjugated anti-TCR β and anti-CXCR4 were purchased from ebioscience (San Diego, CA).

### Establishment of Motile and Non-motile Jurkat or Primary Human and Mouse T cells

**Figure 1:**
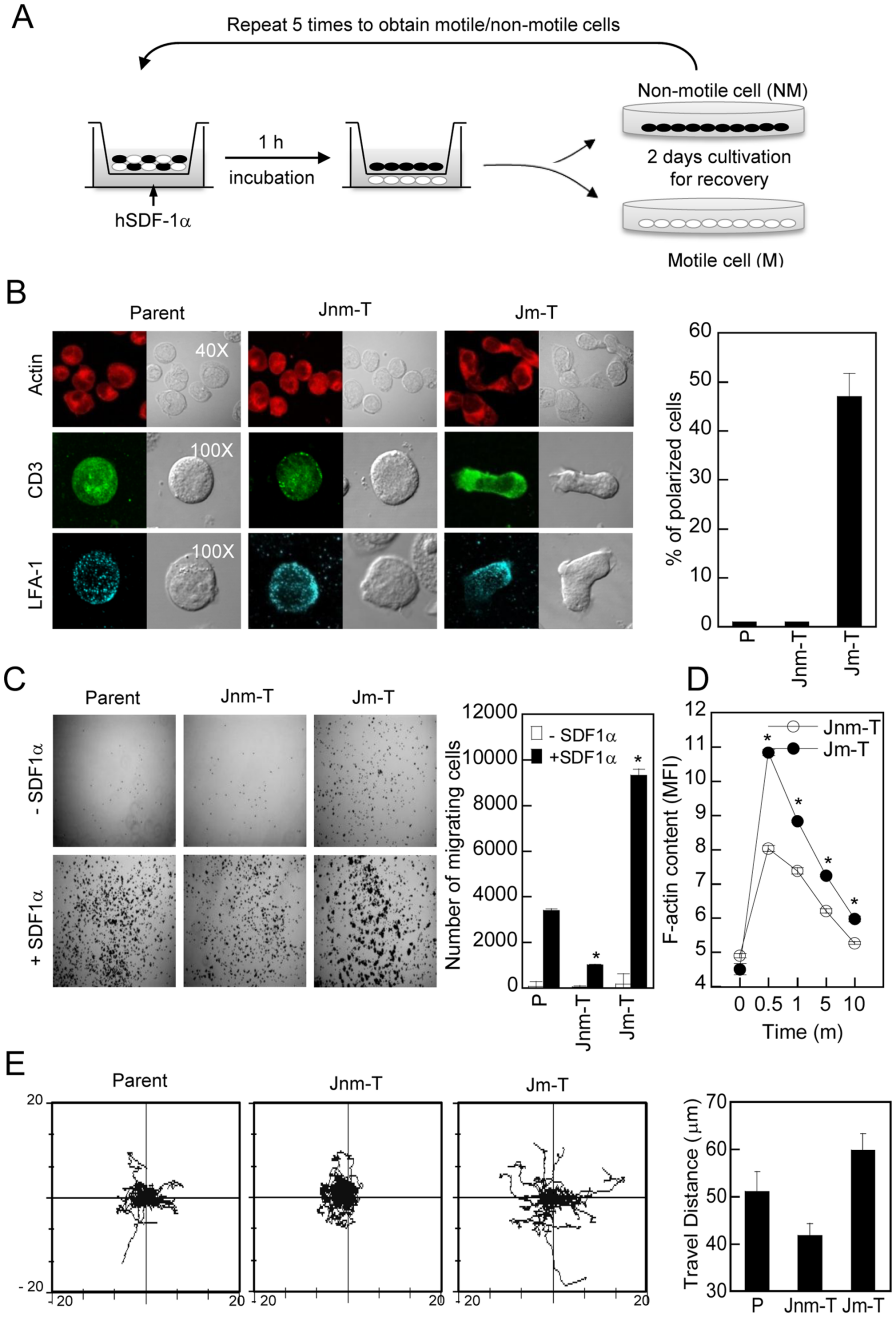
Jurkat motile T (Jm-T) cells have a polarized morphology with high sensitivity to SDF-1α. (A) Schematic diagram for establishment of Jurkat motile T (Jm-T) and Jurkat non-motile T (Jnm-T) cells. (B) Morphologies of parent T, Jm-T, and Jnm-T cells. Polarized cells were quantified by counting >100 cells in each group. (C) Directional migration of parent T, Jnm-T, and Jm-T cells in response to hSDF-1α (50 nM)**.** Cells in the bottom well of the Boyden chamber observed under the microscope (10×) were quantified by flow cytometry. (D) Jnm-T and Jm-T cells (1×10^6^) were stimulated with hSDF-1α. At the indicated time points, the cells were stained with phalloidin-TRITC and the F-actin content was quantified by flow cytometry. The data represent the mean florescent intensity (MFI) and are the mean ± SD of triplicate experiments. *P<0.05 versus Jnm-T cells. (E) Random migratory rate in three groups. Chemokinesis assay was achieved by time**-**lapse confocal microscopy. Cells were allowed to attach and spread on FN-coated glass. Movement of single cell in response to hSDF-1α was tracked and travel distance was calculated using Metamorph.

To establish Jurkat T cell lines with motile and non-motile phenotypes, cells (1×10^6^ cells/ml) were placed in the upper chamber of a 5 µm pore polycarbonate filter (Costar, Cambridge, MA), while hSDF-1α (50 nM) was present in the lower chamber, as described previously [10]. Cells that migrated across the filter into the lower chamber for the first 2 h were collected for establishing Jurkat motile-T (Jm-T) cells, whereas the remaining cells which had not transmigrated for 24 h in the upper chamber were collected for Jurkat non-motile T (Jnm-T) cells. Harvested cells were cultured for 2 days in fresh new media to recover and increase cell numbers. The proportion of two different cell groups was increased by five further rounds of transmigration. Schematic diagram was also shown in Fig. 1A.

To establish human m-T and nm-T cells (Hm-T and Hnm-T), human PBLs were isolated by the method described above and were incubated with PHA (1 µg/ml) and recombinant human IL-2 (10 U/ml) for 3 days for inducing proliferation. To establish mouse m-T and nm-T cells (Mm-T and Mnm-T), mouse T cells were isolated by the method described above, and the cells were incubated with Con A (2 µg/ml), as per the previously described method [11]. Separation of human or mouse m-T and nm-T cells were also performed as per the method described for establishing Jm-T and Jnm-T cell lines. During the separation, primary T cells were maintained with rIL-2 (10 U/ml). Cell viability was assessed by performing the trypan blue exclusion method, and over 90% of the viable cells were used for experiments.

### Immunofluorescence and Confocal Imaging Analysis

For live-time analysis, cells were stained with anti-CD3-Fab-cy3 or LFA-1-Fab-cy3 for 1 h at 4°C and then incubated for 15–30 min with SEE-pulsed Raji B cells stained with Cell Tracker Green CMFDA (Invitrogen) in a live chamber device. In some cases, cells were incubated for 30 min at 37°C on PLL-coated coverslips (18-mm diameter; Fisher Scientific, Pittsburgh, PA), then fixed with 4% paraformaldehyde in PBS and washed twice with PBS. The cells were permeabilized with 0.1% TritonX-100 in PBS and washed twice with PBS. The cells were then incubated with anti-CD3-Fab-cy3 or LFA-1-Fab-cy3, and phalloidin-TRITC in blocking buffer overnight at 4^o^C, rinsed three times with PBS, and mounted with anti-fade solution (Invitrogen). The slides were examined with a FV1000 confocal laser scanning microscope (Olympus, Tokyo, Japan) equipped with 100× objectives.

### Determination of Cellular F-actin Content

Cells were maintained in serum-free medium for 12 h and incubated with hSDF-1α for the indicated times at 37°C. Reactions were terminated by adding 500 µl of 4% paraformaldehyde. Fixed cells were washed once with PBS and resuspended in PBS containing 1% BSA and 0.25% Triton X-100 for 5 min. After permeabilization, cells were washed, stained for 30 min with TRITC-phalloidin, and analyzed by flow cytometry. The raw amount of F-actin was represented as the mean fluorescence intensity (MFI).

### Cell Migration Assay

Transwell cell migration was assayed using a 96 well Boyden chamber (ChemoTx plate, Neuroprobe, Inc., Gaithersburg, MD) according to the manufacturer’s instructions. The Boyden chamber was assembled with polyvinylpyrrolidone-free polycarbonate filters (3–5- µm pore size). Jm-T and Jnm-T cells (5×10^4^) were added to the upper compartment and 50 nM hSDF-1α containing media were added to the lower compartment and incubated for 2 h at 37°C in a humidified CO_2_ incubator. Cells remaining on the top surface of the filter were removed by suction, and those migrating to the lower chamber were harvested. The bottom well was washed two times with PBS, after the last wash, cells were resuspended in 300 µl of PBS and were analyzed by performing flow cytometry for a fixed period of time (300 s) under constant middle pressure.

Chemokinesis was assessed by directly observing the migrating cells in the PC-R-10 bath flow chamber (Live Cell Instruments) after addition of human or mouse SDF-1α. Differential interference contrast (DIC) images were obtained for 1 h at 30 s intervals using a 60× oil immersion objective on FV1000 confocal microscope (Olympus). To track migration paths, series of images were analyzed using MetaMorph image analysis software (Molecular Devices Corporation, Downingtown, PA).

### T Cell Stimulation

Cells were stimulated with either plate-bound anti-CD3 (OKT3 for human, 2C11 for mouse, 10 µg/ml)/CD28 (2 µg/ml) or PMA (200 nM)/A23187 (1 µM). For superantigen stimulation, cells were incubated with SEE (1 µg/ml)-pulsed Raji B cells.

### RT-PCR and Real-time qRT-PCR

Total RNA was isolated from cells with TRIzol reagent and reverse-transcribed by using RT-PreMix (iNtRON Biotechnology). PCR was performed with the following primers (the respective forward and reverse pairs are indicated): human *IL-2*, 5′-CACGTCTTGCACTTGTCAC-3′ and 5′-CCTTCTTGGGCATGTAAAACT-3′; human *GAPDH*, 5′-CGGAGTCAACGGATTTGGTCGTAT-3′ and 5′-AGCCTTCTCCATGGTGGTGAAGAC-3′; human *c-Rel*, 5′-CGAACCCAATTTATGACAACCG-3′ and 5′-TTTTGTTTCTTTGCTTTATTGCCG-3′; human *p65*, 5′-TGGATGGACAGGCGTTG-3′ and 5′-AGACAGGACCTCTGAGAAA-3′. The amplification profile was composed of denaturation at 94°C for 30 s, annealing at 60°C for 20 s, and extension at 72°C for 40 s. The 30 cycles were preceded by denaturation at 72°C for 7 min. Total RNA was isolated and cDNA was synthesized. PCR amplification was performed in DNA Engine Opticon1 (MJ Research) for continuous fluorescence detection in a total volume of 10 µl containing 1 µl of cDNA/control and gene-specific primers by using SYBR Premix Ex *Taq* (Takara Bio). The mRNA levels of the target genes, relative to *GAPDH*, were normalized by using the following formula: relative mRNA expression  = 2^−(ΔCt of target gene − ΔCt of GAPDH)^, where Ct is the threshold cycle value. In each sample, the expression of the gene being analyzed was normalized to that of *GAPDH* and described as the relative mRNA levels to GAPDH or % of maximum.

### ELISA

Cells (1×10^6^) were stimulated as described earlier. After 6–24 h, the amounts of IL-2 in supernatants from three replicas for each condition were determined by ELISA with Duo Set Human or Mouse ELISA kits for IL-2 (R&D Systems).

### Conjugation Assay

Raji B cells were stained with Cell Tracker Orange CMRA (Molecular Probes, Invitrogen) according to the manufacturer’s directions, incubated in the presence or absence of superantigen (SEE for human, 1 µg/ml) for 1 h, washed, and resuspended at a density of 1×10^6^ cells/ml in RPMI. Jm-T and Jnm-T cells were stained with Cell Tracker Green CMFDA (Molecular Probes, Invitrogen) and resuspended at a density of 1×10^6^ cells/ml in RPMI. For T cell–APC conjugation, equal volumes of T cells and APCs were mixed together and incubated at 37°C for 30 min. The relative proportion of red, green, and red-green populations was determined by a Coulter EPICS XL flow cytometer (Beckman Coulter). The number of gated events counted per sample was at least 10,000.

### TCR Internalization Assay

Cells (1×10^6^) were stimulated with anti-CD3/28 antibody for indicated time and stained with PE-conjugated anti-TCRαβ mAb (Invitrogen) at 4°C for 1 h. Cells were then washed with cold PBS to remove unbound antibody and surface-TCRαβ ﬂuorescence was measured by Coulter EPICS XL ﬂow cytometer (Beckman Coulter). The rate of internalization was quantified by following the equation: mean ﬂuorescence intensity (MFI) at 0 h of unstimulated condition – MFI at each time point of unstimulated or stimulated conditions.

### Western Blotting

Cells were lysed in Triton X-100 lysis buffer containing Tris-HCl at pH 7.4 (20 mM), NaCl (150 mM), 1 tablet of Complete protease inhibitors, and phosphatase inhibitors (cocktails I and II). The lysates were centrifuged at 20,000 g for 25 min at 4°C, and the supernatant was eluted with SDS sample buffer (100 mM Tri-HCl, pH 6.8; 4% SDS; 20% glycerol with bromophenol blue) and heated for 5 min. The proteins were separated through 10% SDS-PAGE gels and transferred onto a nitrocellulose membrane (Amersham) by means of Trans-Blot SD semidry transfer cell (Bio-Rad). The membrane was blocked in 5% skim milk for 1 h at room temperature, rinsed, and incubated with the intended antibodies in TBS containing 0.1% Tween-20 (TBS-T) and 3% skim milk for 2 h at room temperature. Excess primary antibody was then removed by washing the membrane four times in TBS-T. The membrane was then incubated with 0.1 µg/ml peroxidase-labeled secondary antibody (anti-rabbit or anti-mouse) for 2 h at room temperature. After three washes in TBS-T, bands were visualized by using ECL western blotting detection reagents (iNtRON Biotechnology) and exposed onto X-ray film.

### Luciferase Promoter Assay

Cells (2×10^6^) were transfected with 100 µl of Amaxa’s Nucleofector solution (Amaxa, Cologne, Germany) containing 3 µg of pGL3-NF-AT, pGL3-NF-κB, and pGL3-IL2 Luc plasmids with pRL-TK. After 24 h, transfected cells (2×10^6^) were stimulated with anti-CD3/28 for 12 h. Cells were harvested and lysed in a lysis buffer (Promega, Madison, WI) and proteins were extracted by a freeze-thaw cycle, and cellular debris was removed by centrifugation at 4°C for 20 min. Luciferase activity was measured with a Centro LB 960 Luminometer (Berthold Technologies, Germany) according to the manufacturer’s instructions. Reporter activity was presented as fold induction of Luciferase activity over that of control cells.

### Statistics

The mean values were calculated from data taken from at least three (usually three or more) separate experiments conducted on separate days. Where significance testing was performed, an unpaired Student’s *t*-test was used. We considered differences between groups significant at *P*<0.05.

## Results

### Jm-T Cells Showed Polarized Shapes with High Sensitiveness to Chemokine

*As explained in Materials and Methods, Jurkat* motile T (Jm-T) or non-motile T (Jnm-T) cells were established using a modified transwell selection system (Fig. 1A). To characterize each group of isolated cells, we first assessed morphological changes as well as distribution of surface proteins such as CD3 and LFA-1 on established T cell lines. Jm-T cells appeared as an elongated form with a polarized shape along with the asymmetric distribution of CD3 and LFA-1, while Jnm-T cells were round and unpolarized (Fig. 1B). To assess the ability of Jm-T cells to respond to chemokine, we investigated directional migration and motility by using Boyden chamber assays. As shown in Fig. 1C, Jm-T cells migrated three times more than parent cells. In contrast, Jnm-T cells migrated toward SDF-1α approximately 0.3-fold slower than parent cells did. Furthermore, we found a significant increment in F-actin levels by SDF-1α in Jm-T cells (Fig. 1D). To analyze the random migratory behavior in each group, *cells were recorded* by *time* **-** *lapse confocal microscopy* at 10-sec intervals, and an overlay of migratory T cell tracks from at least 40 individual cells was created. Furthermore, we measured the average *travel distance* of those populations (Fig. 1E). Compared with Jnm-T cells, Jm-T cells showed high motility in response to hSDF-1α. *Taken together, these results suggest that our experimental system is well established for further characterization of* motile and non-motile T *cells.*

### Jm-T Cells Showed Higher Responsiveness to Activation

**Figure 2:**
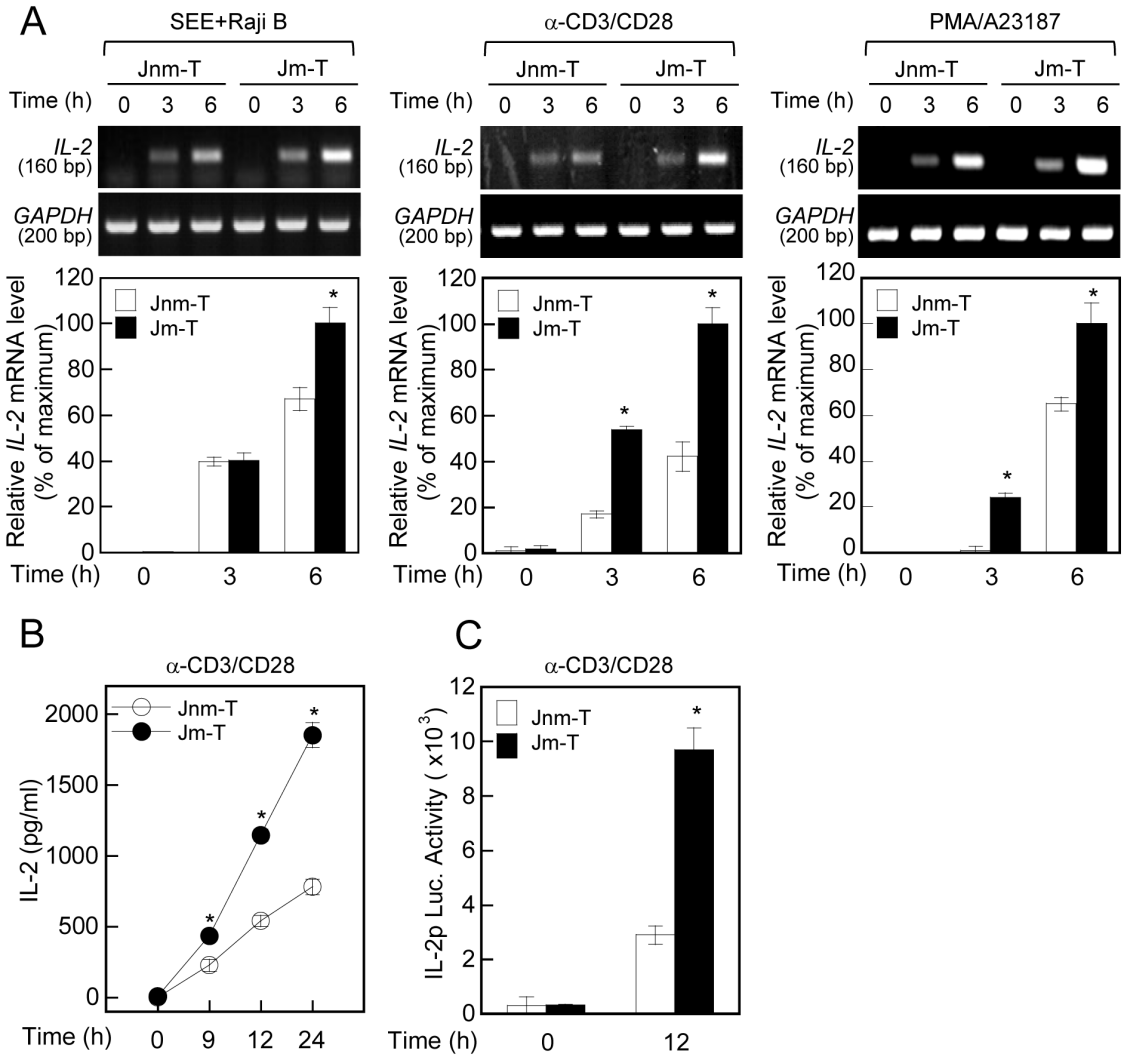
Jm-T cells show higher responsiveness to T cell activation than Jnm-T cells do. (A) Jm-T and Jnm-T cells (1×10^6^) were stimulated with SEE-pulsed Raji B cells, anti-CD3/28, or PMA/A23187 for the prescribed time points. *IL-2* mRNA levels were assessed by RT-PCR (*top*) and real-time qPCR (*bottom*). The results are the mean ± SD of triplicate experiments. (B) Jm-T and Jnm-T cells (1×10^6^) were stimulated with anti-CD3/28 for the prescribed time points, and the cytokine productions (IL-2) were measured by ELISA. *P<0.05, as compared with Jnm-T cells. The results are the mean ± SD of triplicate experiments. (C) Cells (2×10^6^) were transfected with pGL3-IL-2 vector with pRL-TK. The cells were then stimulated for 12 h with anti-CD3/28. The luciferase activities were measured by luminometer. Results are expressed as mean ± SD of three independent experiments.

We investigated whether the differential motility and chemokine response observed in the two cell lines had any correlation to T cell activation. Compared with Jnm-T cells, Jm-T cells showed a significant increase in *IL-2* mRNA levels after stimulation with anti-CD3/28 or incubation with SEE-pulsed Raji B cells, and even treatment with PMA/A23187 (Fig. 2A). To test whether the increase in *IL-2* mRNA expression in Jm-T cells was correlated with the level of protein and promoter activity, we also performed an ELISA and luciferase activity assay. As shown in Fig. 2B and 2C, IL-2 secretion and IL-2 promoter activity in Jm-T cells treated with anti-CD3/28 were significantly higher than those in Jnm-T cells. Taken together, these results demonstrate that Jm-T cells had a better activation capability in response to activation signals than did Jnm-T cells.

### Jm-T and Jnm-T Cells showed Similar Early Events Initiated from the Cell Membrane during T cell Activation

**Figure 3:**
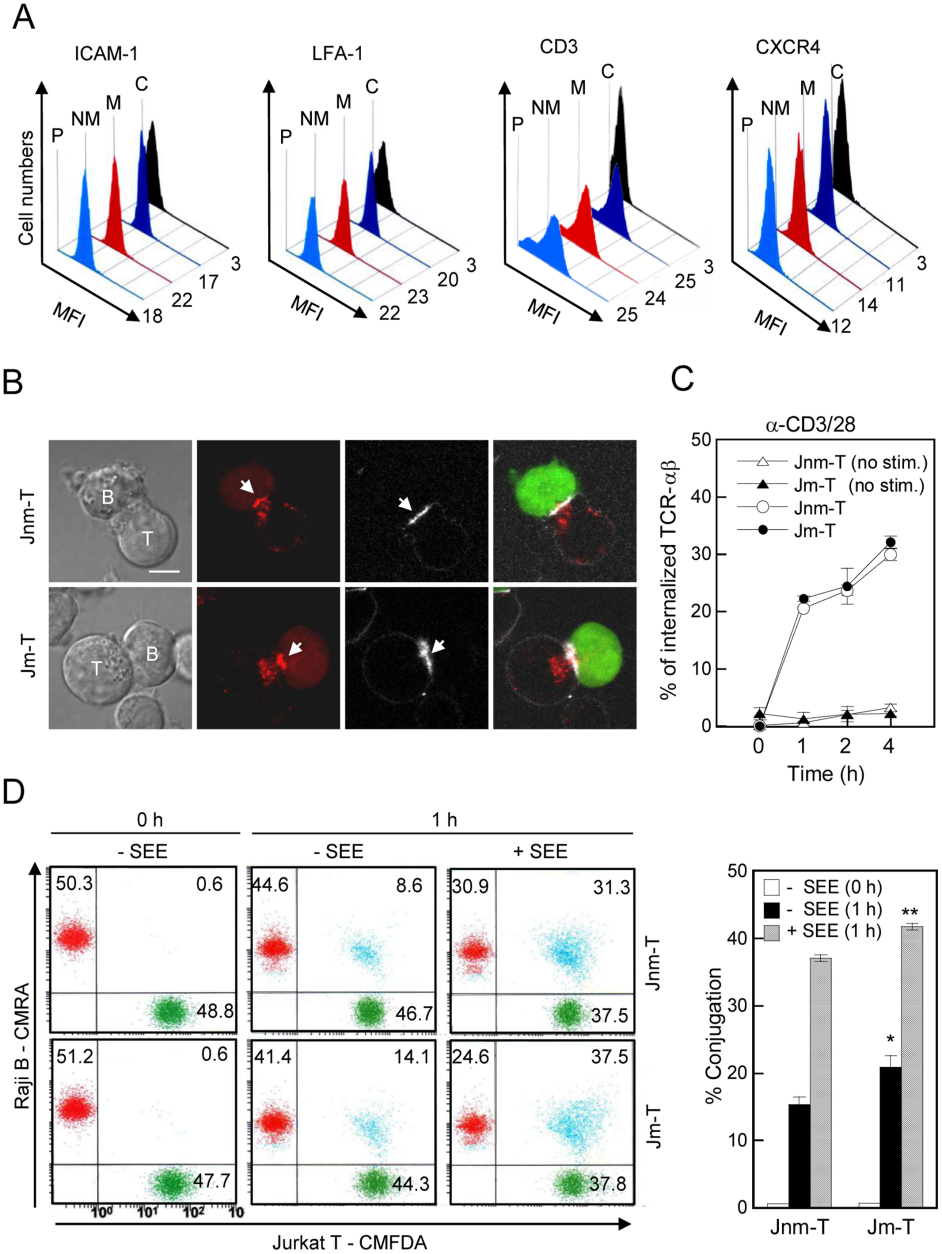
The early events in the cell membrane are not a critical factor to determining hyperactivation in Jm-T cells. (A) Jm-T and Jnm-T cells at a resting state were stained with ICAM-1, LFA-1, CD3 ε-chain, and CXCR4 and analyzed by flow cytometry. C, isotype (IgG) control of parent Jurkat T cells; M, Jm-T; NM, Jnm-T; P, parent Jurkat T cells. The data are representative of four independent experiments. (B) Jm-T and Jnm-T cells (2×10^5^) were stained with anti-CD3 (cy5) or anti-LFA-1 (cy5) Fabs and then incubated with SEE-loaded Raji B cells stained with green cell tracker. The data are representative of four independent experiments. Bar, 10 µm. Arrow heads represent the accumulated molecules at the immunological synapse. (C) Jm-T and Jnm-T cells were stimulated with anti-CD3/28 antibody at 37°C for 1, 2, and 4 h. Cells were then washed and stained with TCR αβ at 4°C for 1 h. The rate of internalization was quantified as described in Materials and Methods. (D) Jm-T and Jnm-T cells (2×10^5^) were incubated with Raji B cells in the presence or absence of SEE (1 µg/mL). A representative conjugate formation profile with T cells and Raji B cells and the percentage of double-positive cells (blue) are shown in the flow cytometric plot and bar graph, respectively. The results are the mean ± SD of triplicate experiments. *P<0.05 as compared with -SEE; **P<0.05 as compared with +SEE.

*To determine whether* higher responsiveness to activation in Jm-T cells is caused by different early events initiated from the cell membrane, we first investigated the surface expression of ICAM-1, LFA-1, CD3ε chain and CXCR4 in resting state of each cell line. As shown in Fig. 3A, surface expression of the indicated proteins did not significantly change in either cell line. *Many T cell-regulating molecules are located in a specialized junctional structure referred to as the immunological synapse during T cell contact with APCs* [*12*] *. We next examined the* mature immunological synapse by measuring the clustering of membrane proteins, including CD3, a key molecule of the c-SMAC, and LFA-1, a p-SMAC molecule; however, the clustering pattern of CD3 and LFA-1 in the SMAC was similar between the groups (Fig. 3B). Furthermore, activation of T cells also leads to the internalization of TCRαβ, we tested TCRαβ internalization after activation. As shown in Fig. 3C, no significant difference in CD3/28-induced TCRαβ internalization was observed between the groups. *On the other hands, immunological synapse formation and maintenance are required for T cell activation* [*13*] *. Therefore, we performed a conjugation* assay after incubation with SEE-pulsed or un-pulsed Raji B cells. Interestingly, Jm-T cells slightly increased T-B conjugates regardless of SEE (Fig. 3D), suggesting that increased F-actin contents and rapid F-actin polymerization in Jm-T cells (Fig. 1D) may affect the increased stabilization of immunological synapse between T cells and APCs. Collectively, these results suggest that the initial membrane events for T cell activation are not involved in the hyper-activation of Jm-T cells, but rather, signaling pathways that mediate rapid and dynamic actin rearrangement may affect the hyper-activation of Jm-T cells.

### PI3K-Akt Pathway is Constitutively Activated and NF-κB Activity is Consequently Higher in Jm-T Cells

**Figure 4:**
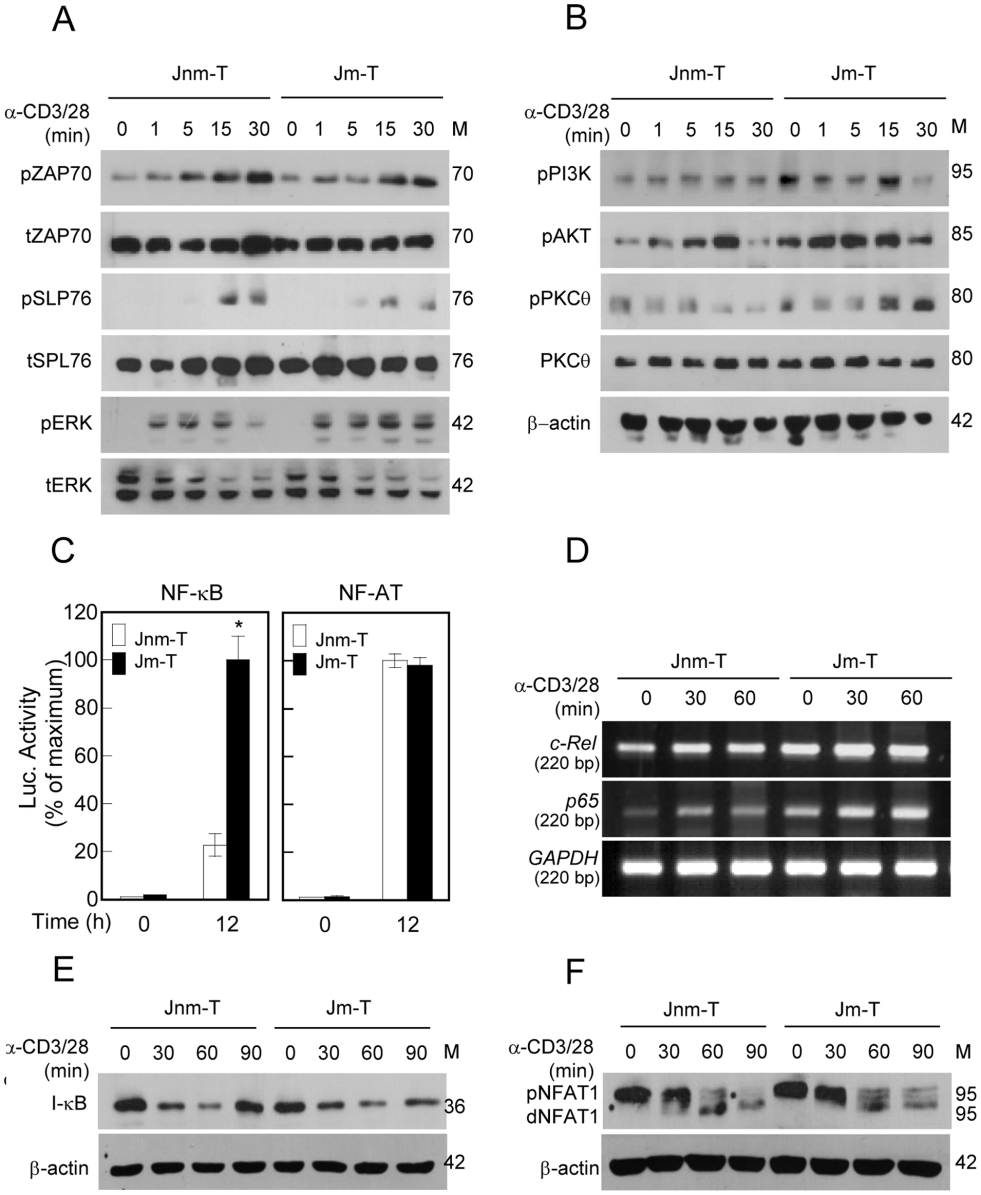
Jm-T cells specifically show higher NF-κB activity through the PI3K-Akt-dependent axis in T cell signaling. (A and B) Jm-T and Jnm-T cells (5×10^6^) were stimulated with plate-bound anti-CD3 for the prescribed time. The cell lysates were immunoblotted for the phosphorylated and total forms of ZAP70, SLP76, ERK, PI3K, Akt, and PKC-θ. The data are representative of at least three independent experiments. (C) Jm-T and Jnm-T cells (2×10^6^) were transfected with pGL3-NF-κB or NF-AT vector with pRL-TK and stimulated for 12 h with anti-CD3/28. The luciferase activities were measured by luminometer. Results are expressed as mean ± SD of three independent experiments. (D) Jm-T and Jnm-T cells (1×10^6^) were stimulated with anti-CD3/28 for the prescribed time. *c-Rel* and *p65* mRNA levels were assessed by RT-PCR. The results are the mean ± SD of triplicate experiments. (E and F) Jm-T and Jnm-T cells (2×10^6^) were stimulated with plate-bound anti-CD3 for the prescribed time. The cell lysates were immunoblotted for I-κB and NF-AT. The data are representative of at least three independent experiments.

To investigate the differences in intracellular signal transduction processes that are related to the higher production of IL-2 in Jm-T cells between the two groups, we next determined phosphorylation of downstream signaling molecules after stimulation with TCR/CD3. Contrary to our expectations, TCR-proximal–signaling molecules, such as ZAP70 and SLP76, were not significantly phosphorylated in Jm-T cells compared with those in Jnm-T cells, although phospho-*ERK* level was slightly enhanced in Jm-T cells *(* *Fig. 4A* *). We next investigated* phosphoinositide3-kinase *(PI3K)-Akt signaling, which is known to be triggered by CD28 receptor engagement* [*14*]. Activation of PI3K-Akt is also a robust signaling event shared by TCR and co-stimulatory receptors as well as most chemokine receptors, contributing to several aspects of T-lymphocyte activation and migration by regulation of actin cytoskeleton reorganization and other components of the general migratory machinery [15], [16]. Interestingly, PI3K and Akt were constitutively activated even in the resting state and highly phosphorylated in response to anti-CD3/28 stimulation in Jm-T cells. Phosphorylation of PKC-θ, a downstream target protein of Akt, was also more enhanced in Jm-T cells than in Jnm-T cells (Fig. 4B). Phosphorylation and translocation of PKC-θ is a key step for NF-κB activation [17], [18]. We found that NF-κB promoter activity was significantly increased in Jm-T cells by TCR/CD3 activation (Fig. 4C). In contrast, interestingly, no significant difference in NF-AT promoter activity was observed between Jm-T and Jnm-T cells (Fig. 4C). Along this line, a considerable increase in the expression of *c-Rel* and *p65*, NF-κB subunits, mRNA was observed in Jm-T cells in response to anti-CD3/28 stimulation (Fig. 4D). The activation of NF-κB requires phosphorylation and degradation of I-κB, a component of NF-κB that binds to NF-κB and blocks the nuclear translocation of NF-κB. Although TCR-mediated I-κB degradation was detected in both Jm-T and Jnm-T cells, it was prolonged up to 90 min after anti-CD3/28 stimulation in m-T cells (Fig. 4E). In contrast, the NF-AT phosphorylation state in Jm-T cells was similar to that observed in Jnm-T cells. Taken together, these results demonstrate that Jm-T cells are more strongly activated than Jnm-T cells through activation of the PI3K-Akt-NF-κB axis in T cells. In addition, these results suggest that T cells can be subcategorized on the basis of their intrinsic migratory capacity in relation to their activation.

### Motile and Non-motile Primary T cells also Revealed Higher Responsiveness to T cell Stimulation

**Figure 5:**
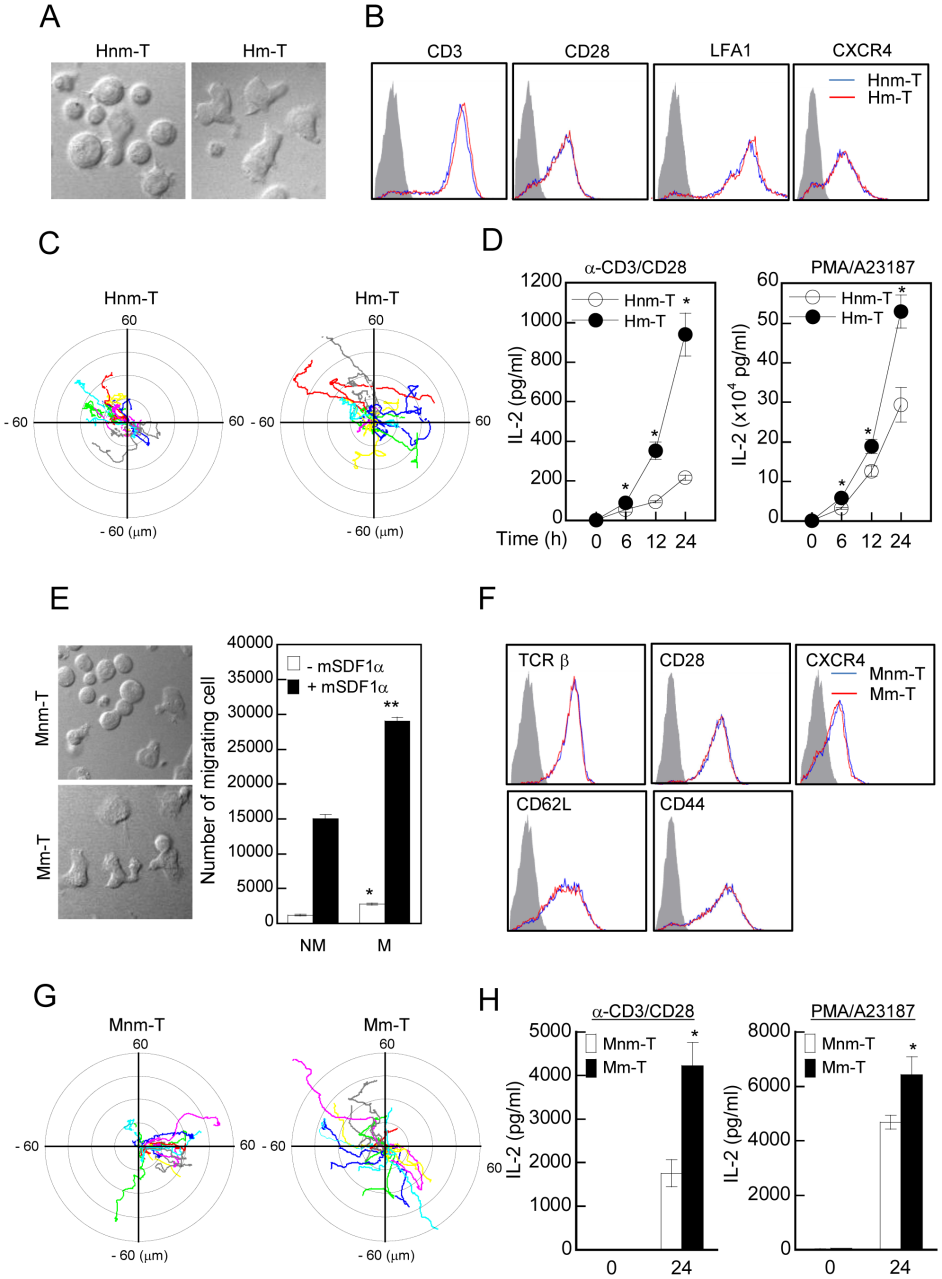
Motile primary T cells show higher responsiveness to T cell activation than non-motile primary T cells do. (A) Morphologies of human motile and non-motile primary T cells. (B) Surface expression of CD3, CD28, CXCR4, and LFA-1 was determined by flow cytometry. The data are representative of three independent experiments. The shaded histogram represents isotype control for antibody, blue line represents Hnm-T, and red line represents Hm-T cells. The data are representative of four independent experiments. (C) Random migratory rate in two groups. Chemokinesis assay was achieved by time**-**lapse confocal microscopy. Cells were allowed to attach and spread on ICAM1-coated glass. Movement of single cell in response to hSDF-1α was tracked by using Metamorph. (D) Hm-T and Hnm-T cells (1×10^6^) were stimulated as described in Fig 2, and the cytokine productions (IL-2) were measured by ELISA. *P<0.05, as compared with Hnm-T cells. (E) Morphologies and directional migration of Mm-T and Mnm-T cells. Cells in the bottom well of the Boyden chamber were quantified by flow cytometry. *P<0.05, as compared with Mnm-T cells in the absence of SDF1α; **P<0.05, as compared with Mnm-T cells in the presence of SDF1α. (F) Surface expression of TCR β, CD28, CXCR4, CD44, and CD62L was determined by flow cytometry. The data are representative of three independent experiments. (G) Random migratory rate in two groups. Chemokinesis assay was achieved by time**-**lapse confocal microscopy. Cells were allowed to attach and spread on ICAM1-coated glass. Movement of single cell in response to mSDF-1α was tracked by using Metamorph. (H) Mm-T and Mnm-T cells (1×10^6^) were stimulated as described in Fig 2, and the cytokine productions (IL-2) were measured by ELISA. *P<0.05, as compared with Mnm-T cells.

To further corroborate whether these biological characteristics of Jurkat T cells are reproducible in primary T cells, we established human and mouse motile and non-motile primary T cell lines in the same way. Similarly to the Jurkat m-T cells, human and mouse motile T cells (Hm-T and Mm-T cells) also showed an elongated phenotype with a polarized shape and higher responsiveness to chemokine (Fig. 5A and 5E). In addition, there were basically no differences in terms of surface expression levels of the indicated proteins (Fig. 5B and 5F). We next investigated random migration by using live chamber. As shown in Fig. 5C and 5G, both human and mouse motile T cells showed higher motility in response to SDF-1α than the non-motile T cells. We finally examined whether the differential motility and chemokine response observed in the two cell lines have any correlation to T cell activation. Compared with non-motile T cells, motile T cells showed a significant increase in IL-2 production after stimulation with anti-CD3/28 or treatment with PMA/A23187 (Fig. 5D and 5H). Taken together, these results demonstrate that motile T cells had a better activation capability in response to activation signals than did non-motile T cells.

## Discussion

In this study, we found that T cells can be subcategorized based on their inherent migratory capacity rather than the surface expression of specific differentiation-related markers. We characterized features of motile and non-motile T cells with respect to T cell activation. Interestingly, we observed that motile T cells exhibited elevated NF-κB activity through the PI3K-Akt signaling pathway and increased secretion of the cytokine IL-2 in response to T-cell activation signals.

Chemokines, in particular SDF-1α, signal through CXCR4, a G-protein-coupled chemotactic receptor, and induce directional F-actin polymerization in the lamella region of migrating T cells [19]. Downstream of the CXCR4, the regulation of F-actin polymerization depends on PI3K activation and PtdIns(3,4,5)P3 production, followed by the activation of protein kinase B (Akt/PKB) and GTPases [20]. These events are known to be coupled with morphological changes from round and symmetrical to a polarized or elongated phenotype and asymmetrical shape. In accordance with this, the motile T cells that we established in the current study showed typical features of motile lymphocytes that have thus far been well characterized.

T cells are activated by several signaling pathways initiated from TCR and a co-receptor. The main signaling event is phosphorylation of immunoreceptor tyrosine-base activation motifs (ITAMs) on the cytosolic side of the TCR/CD3 complex by lymphocyte protein-tyrosine kinase (Lck) [21]. ζ-Chain-associated protein kinase (ZAP-70) is recruited to the TCR/CD3 complex where it is activated, promoting recruitment and phosphorylation of downstream adaptor or scaffold proteins [22]. A downstream second messenger, diacylglycerol (DAG), activates PKC-θ and the MAPK/ERK pathway, both promoting transcription factor NF-κB activation [23], whereas IP_3_ triggers the release of Ca^2+^ from the ER, which promotes the entry of extracellular Ca^2+^ into cells and finally promotes *IL-2* gene transcription through the transcription factor NF-AT [24], [25], [26]. The other additional signal CD28 simultaneously induces signal transduction by binding to PI3K, a family of complex enzymes with multifunctional roles [27], [28], [29], [30], [31]. Interestingly, CD28 signaling has previously been associated with the regulation of integrin-mediated T-cell adhesion as well as cytoskeletal reorganization [32], [33]. In our present study, Jurkat motile T cells showed constitutive activation of PI3K and AKT, thereby suggesting that cellular migratory capacity, in part, clearly overlaps with activation capacity through the same signaling pathway in T cells. In this regard, it is well known that PI3K is activated by receptor and non-receptor protein tyrosine kinases and by G protein-coupled receptors on chemokine stimulation of migrating cells [34]. This CD28-PI3K pathway ultimately triggers a number of downstream signaling molecules such as Akt and PKC-θ and plays a critical role in driving cytokine production, proliferation, and survival. In our study, phosphorylation of PI3K was dramatically increased in Jurkat motile T cells; consequently, Akt, a downstream target of PI3K, was also highly phosphorylated. In contrast, the activation profiles of ZAP70 and SLP76, molecules involved in TCR-proximal-signaling pathway, were similar between Jurkat motile and non-motile T cells. This result clearly suggests that hyperactivation of motile T cells is induced not by the TCR-ZAP70 but by the CD28-PI3K pathway. Consequently, high PKC-θ activity by the PI3K-Akt pathway might induce NF-κB activation and contribute to IL-2 production.

There might be another reason that motile T cells show better activation capability than non-motile T cells do. Motile T cells have shown a polarity and that this polarized microenvironment provide sensitivity against stimulants. Furthermore, the leading edge of motile T cells might be more effective at engaging or aggregating receptors to generate an adequate signal; therefore, we could hypothesize that higher cytokine production in motile cells might be caused by polarized molecules that effectively and sensitively induce signals from the membrane. Unexpectedly, in addition to TCR-mediated signaling, IL-2 cytokine was produced at high levels by PMA/A23187 stimulation. These data suggest that not only polarity but also intracellular signaling in motile T cells is distinct from that in non-motile T cells. Indeed, expressions of NF-κB components, *c-rel* and *p65*, were intrinsically higher in motile T cells than in non-motile T cells.

In general, T cell motility is required for migration within the T cell zone and for making contact with APCs. Antigen recognition may depend on a stochastic process in which chance encounters take place between T cells and APCs. Therefore, two populations of T cells, phenotypically distinct with regard to their response to chemokine and migration capacity, may affect the initial screening for the antigen in the SLOs. In the current study, we added the concept that motility of T cells is also linked to the T cell activating potential even after antigen recognition. In this regard, it will be interesting to examine which of the T cell subsets shows similar or identical phenotypes of motile T cells in vivo. In our recent unpublished results, interestingly, we have noticed that “antigen-experienced” memory T cells are highly dynamic compared to naïve T cells, even in the absence of chemokines. Although memory T cells respond more vigorously to stimulation and they are more sensitive to low doses of antigen than naive T cells, the molecular basis of this increased sensitivity remains unclear. Our current results suggest that rapid and strong response of memory T cells to the antigen may also be related to the motility of T cells. We, therefore, suggest that this motile population is a sort of specific subgroup and think that these two groups may have different intrinsic gene profiles. Global gene expression profiling is now carried out with motile and non-motile T cells with or without T cell stimulation. On the other hand, motility may permit the escape of T cells from lymph nodes and be essential for their entrance into peripheral tissues to perform effector function after T cell activation. Our preliminary results of primary human or mouse T cells additionally demonstrated that motile T cells showed enhanced spontaneous transendothelial migration, suggesting that motile T cells in vivo may have infiltrating capacity into peripheral tissues or even inflammatory sites.

The studies presented here are the first to show that motile T cells had a better activation capability in response to activation signals than did non-motile T cells. The PKC-θ-NF-κB pathway followed by the PI3K-Akt axis is highly activated in m-T cells. In addition, these in vitro findings provide further insight into the reduction of inflammation by controlling recruitment and activation of effector T cells by tightly controlling PI3K-Akt activity and NF-κB signaling.
